# Disease-associated immune cell endotypes in anti-MDA5-positive dermatomyositis using unbiased hierarchical clustering

**DOI:** 10.3389/fimmu.2024.1349611

**Published:** 2024-03-12

**Authors:** Ruru Guo, Yang Yang, Liyang Gu, Xinyu Li, Yiyangzi Ma, Xuesong Liu, Liangjing Lu

**Affiliations:** ^1^ Department of Rheumatology, Ren Ji Hospital, Shanghai Jiao Tong University School of Medicine, Shanghai, China; ^2^ Department of Ultrasound, Renji Hospital, School of Medicine, Shanghai Jiao Tong University, Shanghai, China

**Keywords:** dermatomyositis, MDA5, endotype, interstitial lung disease, immune cell

## Abstract

**Objective:**

Clinical and prognostic features of Anti-MDA5-Positive Dermatomyositis (MDA5+ DM) are diverse. This study aimed to examine the peripheral immune cell profiles of patients with MDA5+ DM, identify disease endotypes related to the heterogeneous manifestations and prognosis, and guide individualized therapy regimen.

**Methods:**

This inpatient cohort included 123 patients with MDA5+ DM. Unsupervised hierarchical clustering analysis was used to derive disease endotypes from the circulating immune cell profiles on admission. Clinical symptoms, laboratory test results, inpatient treatments, and disease outcomes were then analyzed among the identified endotypes.

**Results:**

Three disease endotypes in MDA5+ DM were identified from peripheral immune cell profiles. Endotype1 had the highest percentages of CD4^+^ T cells and monocytes, and the lowest percentage of neutrophils; Endotype2 had the highest percentage of B cells; Endotype3 had the highest percentage of CD8^+^ T cells and NK cells. Clinical and prognostic heterogeneity of the endotypes were revealed. Endotype1 had the lowest 3-month mortality with the high incidence of periungual capillary changes. Endotype2 and Endotype3 had higher prevalence of rapidly progressive interstitial lung disease (RPILD) and mortality at 3 months than Endotype1. Meanwhile, Endotype3 had higher pneumocystis jiroveci and CMV viremia cases with significantly elevated of activated CD8^+^ T cells and multiple cytokines than Endotype1.

**Conclusion:**

Clustering analysis of peripheral immune cell profiles identified three different endotypes in MDA5+ dermatomyositis. Endotpye2 and 3 showed higher RPILD, 3-month mortality, pneumocystis jiroveci and CMV viremia.

## Introduction

Anti-MDA5 positive dermatomyositis (MDA5+ DM) is a type of idiopathic inflammatory myopathy with positive MDA5 autoantibody, involvement of multiple organs, heterogeneous clinical spectrum of manifestations, and high mortality (1, 2). The high mortality rate means that MDA5+ DM is a substantial challenge for clinical rheumatologists. Multiple prognostic factors have been found for this myopathy (3).

Rapidly progressive interstitial lung disease (RPILD) is the major prognostic factor that can divide patients into those with and without RPILD, leading to different short-term outcomes and therapeutic needs. For MDA5+RPILD, initiating a combined immunosuppressive therapeutic regimen early in the disease course should be considered the first-line therapy, which improves morbidity and mortality (4, 5). Several treatment protocols included the combination of high-dose glucocorticoids and calcineurin inhibitors or a triple therapy adding intravenous cyclophosphamide (5), while plasma exchange can be added for refractory disease. And tofacitinib and rituximab might have a role in the therapeutic armamentarium of this challenging to treat (4, 6, 7). These protocols significantly improved early-stage patients, whereas the 6-month mortality rate remained at around 40% (3, 8). Disease outcomes and treatments remain unsatisfactory. Clinical and prognostic heterogeneity further exacerbate the complexity and uncertainty in the development of novel treatment options.

To address this heterogeneity, the identification of clinical phenotypes and disease endotypes is required. Three distinct clinical phenotypes have been proposed, according to the predominance of pulmonary, skin-articular, or vascular symptoms in MDA5+ DM (9). However, endotype identification is still lacking. Diseases such as asthma and osteoarthritis are more advanced than MDA5+ DM in disease endotyping (10–12). Unlike phenotypes, endotypes are subtypes that are classified based on cellular, immunological, molecular, or genetic biomarkers rather than on clinically observable symptoms (9, 10). The identification of endotypes could provide deeper insights into the underlying pathological mechanisms to guide clinical management and therapeutic development. Although the aetiology and pathology remain unclear, increasing evidence suggests that multiple factors, including T cells, B cells, neutrophils and macrophages, are implicated in the pathophysiology of MDA5+DM (2). The inclusion of calcineurin inhibitors (cyclosporine A, tacrolimus) in several immunosuppressive regimens indicates that targeting T cells is an effective means to treat MDA5+ DM (3, 13). In addition, recent studies showed MDA5+ DM patients with co-existing anti-Ro52 antibodies had an increased frequency of RPILD and more aggressive phenotypes, highlighting the breach of B cell tolerance likely contributes to the pathogenesis in MDA5+ DM (13, 14). B cell depletion with rituximab has been used to treat dermatomyositis (15), which could be an alternative treatment for MDA5-DM in severe and refractory cases (2). Studies have also shown that both monocytes and lymphocytes are correlated with different prognoses of MDA5+ DM (16, 17). Lower peripheral CD3^+^T cell counts were independently associated with poorer prognosis in MDA5+DM-RPILD (18), while higher peripheral CD3^+^T cell counts were associated with longer survival. The independent risk factors for RPILD in MDA5+DM patients included lymphopenia, especially decreased levels of CD3^+^CD4^+^ T cells, elevated CD5^-^CD19^+^ B cells (19). But an integrated evaluation and multi-dimensional characterization of immune cell profiles have not been achieved. The peripheral immune cells mentioned above seem to be a simple and practical approach to predicting the prognosis of MDA5+ DM in clinical practice (2). So, we exploratively performed hierarchical clustering analysis based on immune cell profiles (percentages of neutrophils, monocytes, CD3^-^CD16^+^CD56^+^NK cells, CD3^+^CD4^+^ T cells, CD19^+^B cells, and CD3^+^CD8^+^ T cells) in an inpatient cohort, and analyzed the clinical and prognostic features of the identified endotypes to characterize them and to fully depict their clinical and prognostic significance. Thus, our current study may provide new clues for the disease endotype classifications, immunopathogenesis and potential therapeutic targets for MDA5+ DM.

## Methods

### Study cohorts

Clinical data from patients diagnosed with DM from July 2018 to January 2022 were reviewed in our electronic medical record system. A total of 123 MDA5+ DM patients were hospitalized and enrolled according to the Wu W et al. (2021)’s summary of MDA5+DM classification and the 239^th^ ENMC proposed criteria (3, 20). Classification is based on the presence of typical DM rashes (such as Gottron’s sign, heliotrope rash) and a positive MDA5 autoantibody test. Patients with insufficient data or other autoimmune diseases were excluded. Demographic characteristics, clinical symptoms on admission, laboratory results, inpatient treatments, and clinical outcomes were retrospectively collected. The pathomechanism of MDA5+ DM is complex, and several lines of evidence indicate a potential contributing role for T cells, B cells, neutrophils, and natural killer cells (2, 13). Therefore, peripheral simple immune subpopulations were further detected, including T cells, B cells, NK cells. This study was conducted in accordance with the principles of the Declaration of Helsinki. Patient consent was obtained for the collection of research data. Institutional ethics approvals were obtained (approval number: 2017-201).

### Detection of autoantibodies

Immunoblot testing (Euroimmun, Lubeck, Germany) was used to detect MDA5 and other myositis-specific or -associated autoantibodies according to the manufacturer’s instructions as previously described (21). Briefly, the gray-scale value of the antibody band was scanned to obtain semiquantitative results, and gray-scale values of 0 to 5 units/L were defined in the following manner: 11 to 25 units/L as +, 26 to 50 units/L as ++, and >50 units/L as +++.

### Assessment of disease

FLAIR score was assessed based on presence of RPILD, high-resolution computed tomography imaging (HRCT), anti-MDA5 antibody, ferritin, and LDH levels, which has been reported to predict mortality risk in amyopathic dermatomyositis (ADM) (21). Due to the lack of a systematic risk prediction model for MDA5+ DM and the fact that ADM and MDA5+ DM substantially overlap (3), the FLAIR score was calculated as a reference prognostic indicator. RPILD was defined as worsening dyspnea and high-resolution computed tomography progression within 1 month or respiratory failure within 3 months since respiratory symptoms appeared (9). Spontaneous pneumomediastinum and spontaneous intramuscular hemorrhage were evaluated by CT, ultrasound or magnetic resonance imaging. Pneumocystis infection was confirmed using next-generation sequencing. Laboratory data beyond the detection range were recorded with the limit.

### Flow cytometry analysis

The immune cells for the flow cytometry analysis were collected at the time of admission and transported to our testing center within 2 hours. PBMCs were then isolated and analyzed. Peripheral lymphocyte subpopulations were detected with BD Multitest 4-color TBNK reagent. Flow cytometric phenotyping of expression of selected activation markers (CD38 on CD8^+^ cells, HLA-DR on CD3^+^ cells, CD38 and HLA-DR co-expression on CD8^+^ cells) in 33 collected samples were detected with a BD FACS Caliber Flow Cytometer (BD Biosciences, San Diego, CA, USA) as previously described (15). The gating strategies are illustrated in **Supplementary Figures 1**-**2**. The number of lymphocytes used in each flow cytometry analysis was ensured to be above 2500.

### Immunophenotyping features and unsupervised cluster analysis of patients with DM

Principal component analysis (PCA) was conducted to visualize immune cell profile differences between patients with MDA5+ DM and healthy controls. Unsupervised hierarchical clustering analysis was performed to classify patients into different endotypes by the Ward method on Euclidian distances, based on the percentages of neutrophils, monocytes, CD3^-^CD16^+^CD56^+^NK cells, CD3^+^CD4^+^ T cells, CD19^+^B cells (namely, B cells), and CD3^+^CD8^+^ T cells.

### Statistical analyses

Quantitative variables were expressed as mean ± standard deviation, and categorical data were expressed as numbers (percentage). Unpaired t-tests or Mann–Whitney U tests were used between two groups as appropriate. One-way ANOVA or Kruskal-Wallis H tests were used among three groups, while categorical variables were compared using chi-squared or Fisher’s exact tests as appropriate, followed by *post-hoc* tests for specific differences between every two groups. Survival data were analyzed using Kaplan-Meier plots and log-rank tests. GraphPad Prism statistical software version 9.0.0 (GraphPad Software, San Diego, California USA), R version 4.1.1 (R Core Team, Vienna, Austria), and IBM SPSS Statistics for Windows version 26.0 (Armonk, NY: IBM Corp) were used to generate the statistical results. Statistical significance was set at *P* < 0.05.

## Results

### Clinical characteristics of MDA5+ DM at the baseline

The present study included 123 patients with MDA5+ DM (males: 26.02%, average age: 52.31 years) and eighty-eight healthy controls (HC) with a mean age of 52.84 years (males 26.14%). The most common symptom in the cohort was Gottron’s sign (n = 91, 73.9%), and RPILD was detected in 39 cases (31.7%). The average observation time was 245.00 ± 232.20 days, with 31 cases died in three months, and 92 cases continued follow-ups after three months. The treatment regimen at the time of enrollment was summarized in **Supplementary Table 1**. The overall clinical characteristics of MDA5+DM were shown in **Tables 1** and **2**.

**Table 1:** Demographic and clinical characteristics of the three MDA5+ DM Endotypes.

Characteristics	All MDA5+DM (n=123)	Endotype 1 (n = 45)	Endotype2 (n = 42)	Endotype3 (n = 36)	*overall P*-value
Age (years)	52.31 ± 12.07	52.89 ± 11.04	52.83 ± 11.71	50.97 ± 13.84	0.735
Sex (M/F)^a^	32/91	6/39	15/27	11/25	**0.045**
Clinical manifestations					
Shawl sign (n, %)	29 (23.6)	10 (22.2)	13 (31)	6 (16.7)	0.322
Gottron’s sign (n, %)	91 (73.9)	32 (71.1)	32 (76.2)	27 (75)	0.853
Heliotrope rash (n, %)	44 (35.8)	12 (26.7)	19 (45.2)	13 (36.1)	0.196
Mechanic hand (n, %)	39 (31.7)	16 (35.6)	16 (38.1)	7 (19.4)	0.165
V sign (n, %)	38 (30.9)	13 (28.9)	13 (31)	12 (33.3)	0.912
Periungual capillary changes (n, %)^a^	21 (17.1)	12 (26.7)	7 (16.7)	2 (5.5)	**0.043**
Myalgia or muscle weakness (n, %)^b^	38 (30.9)	15 (33.3)	19 (45.3)	4 (11.1)	**0.005**
RPILD (n, %)^c^	39 (31.7)	6 (13.3)	23 (54.8)	10 (27.8)	< **0.001**
Arthralgia (n, %)	35 (28.5)	18 (40)	7 (16.7)	10 (27.8)	0.054
Fever (n, %)	27 (21.9)	9 (20)	7 (16.7)	11 (30.6)	0.310
Spontaneous pneumomediastinum (n, %)^d^	12 (9.8)	0 (0)	7 (16.7)	5 (13.9)	**0.005**
Hoarseness (n, %)	5 (4.1)	3 (6.7)	2 (4.8)	0 (0)	0.376
Spontaneous intramuscular hemorrhage (n, %)	1 (0.8)	0 (0)	0 (0)	1 (2.8)	0.293
Days of follow-up (days)	245.00 ± 232.20	275.00 ± 331.40	208.00 ± 287.8	250.60 ± 355.20	0.626
Laboratory results					
Leukocyte counts (×10^9/L)^e^	7.20 ± 3.38	5.94 ± 2.64	8.21 ± 3.67	7.59 ± 3.43	**0.002**
Ferritin (μg/L)	1142 ± 1178	1048.10 ± 931.12	1391.92 ± 1662.5	968.04 ± 623.69	0.229
LDH (U/L)	357.70 ± 182.70	318.20 ± 106.26	390.55 ± 245.36	368.58 ± 167.89	0.167
CRP (mg/L)^b^	10.95 ± 20.10	8.24 ± 21.42	9.22 ± 10.65	16.61 ± 26.13	**0.016**
ESR (mm/h)	38.63 ± 23.08	37.20 ± 24.47	38.36 ± 22.25	40.75 ± 22.72	0.788
Fibrinogen (g/L)^a^	3.33 ± 1.03	3.04 ± 0.80	3.35 ± 1.07	3.67 ± 1.17	**0.024**
ALT (U/L)	75.16 ± 99.18	77.62 ± 95.13	96.00 ± 132.01	47.78 ± 34.61	0.157
γGT (U/L)^e^	134.00 ± 212.70	78.47 ± 76.43	216.61 ± 333.3	114.46 ± 103.94	**0.017**
ALP (U/L)^e^	91.42 ± 46.51	74.80 ± 20.23	108.27 ± 58.03	95.71 ± 49.09	**0.001**
CK (U/L)	115.60 ± 230.8	89.78 ± 96.50	140.4 ± 259.8	121.9 ± 309.4	0.396
Cr (μmol/L)^a^	51.47 ± 20.23	46.19 ± 9.77	51.28 ± 15.32	58.30 ± 30.79	**0.020**
Urea (mmol/L)^d^	6.17 ± 2.99	4.93 ± 1.36	7.08 ± 3.30	6.81 ± 3.64	< **0.001**
UCR^e^	0.12 ± 0.04	0.11 ± 0.31	0.14 ± 0.05	0.12 ± 0.38	**0.049**
CD4/CD8^b^	2.29 ± 1.40	3.00 ± 1.46	2.66 ± 1.12	1.00 ± 0.46	< **0.001**
Cytomegalovirus DNA above normal (n, %)^d^	13 (10.6)	0 (0)	6 (14.3)	7 (19.4)	**0.003**
Pneumocystis infection (n%)^a^	7 (5.7)	0 (0)	2 (4.8)	5 (13.9)	**0.018**

One-way ANOVA or Kruskal-Wallis H tests were used among three groups, while categorical variables were compared using chi-squared or Fisher’s exact tests as appropriate, followed by post-hoc tests for specific differences between every two groups.

a There is significant difference between Endotype1 and Endotype3.

b There is significant difference: Endotype1 vs Endtoype3, Endotype2 vs Endotype3.

c There is significant difference: Endotype1 vs Endtoype2, Endotype2 vs Endotype3.

d There is significant difference: Endotype1 vs Endtoype2, Endotype1 vs Endotype3.

e There is significant difference between Endotype1 and Endotype2.

RPILD, rapidly progressive interstitial lung disease; LDH, lactate dehydrogenase; CRP, C-reactive protein; ESR, erythrocyte sedimentation rate; ALT, alanine transaminase; γGT, γ-glutamyl transpeptidase; ALP, alkaline phosphatase; CK, creatine kinase; Cr, creatinine; UCR, urea to creatinine ratio; NMR, neutrophil to monocyte ratio.

The P-value reflected the allover difference among the three groups.

The specific differences among every two groups were shown in **Supplementary Table 3**. Values in bold are statistically significant at p < 0.05.

**Table 2:** Inpatient treatment and prognosis of the MDA5+ DM patients.

Characteristics	All MDA5+DM (n=123)	Endotype1 (n = 45)	Endotype2 (n = 42)	Endotype3 (n = 36)	*overall* *P*-value
Inpatient Treatments					
Low-dose steroid^a^	22 (17.9)	6 (13.3)	4 (9.5)	12 (33.3)	**0.002**
Median-dose steroid^b^	86 (69.9)	38 (84.4)	29 (69)	19 (52.8)	
High-dose steroid^c^	15 (12.2)	1 (2.2)	9 (21.4)	5 (13.9)	
DMARDs^a^	103 (83.7)	38 (84.4)	39 (92.9)	26 (72.2)	**0.048**
Biologic DMARDs	18 (14.6)	5 (11.1)	7 (16.7)	6 (16.7)	0.703
Tac^a^	66 (53.7)	21 (46.7%)	29 (69.0%)	16 (44.4%)	**0.047**
CyA	22 (17.9)	10 (22.2%)	7(16.7%)	5 (13.9%)	0.603
CTX	4 (3.3)	3 (6.7%)	0	1 (2.8%)	0.205
MMF	2 (1.6)	1 (2.2%)	0 (0%)	1 (2.8%)	0.748
Jaki	22 (17.9)	9 (20%)	9 (21.4%)	4 (11.1%)	0.445
Thalidomide	10 (8.1)	3 (6.7%)	6 (14.3%)	1 (2.8%)	0.199
Basiliximab	13 (10.6)	4 (8.9%)	6 (14.3%)	3 (8.3%)	0.709
Rituximab	2 (1.6))	1 (2.2%)	0 (0%)	1 (2.8%)	0.748
Tocilizumab	4 (3.3)	1(2.2%)	1 (2.4%)	2 (5.6%)	0.681
Iguratimod	11 (8.9)	2 (4.4%)	4 (9.5%)	5 (13.9%)	0.323
HCQ	10 (8.1)	2 (4.4%)	4 (9.5%)	4 (11.1%)	0.499
No.of sDMARDS ≥ 2	39 (31.7)	11 (24.4)	19 (45.2)	9 (25)	0.067
Prognosis					
Progression of ILD^bc^ during hospitalization	43 (34.9)	6 (13.3)	21 (50)	16 (44.4)	**0.001**
Inpatient death^bc^	25 (20.3)	2 (4.4)	11 (26.2)	12 (33.3)	**0.003**
3-month mortality^bc^	31 (25.2)	5 (11.1)	14 (33.3)	12 (33.3)	**0.024**

One-way ANOVA or Kruskal-Wallis H tests were used among three groups, while categorical variables were compared using chi-squared or Fisher’s exact tests as appropriate, followed by post-hoc tests for specific differences between every two groups.

a There is significant difference between Endotype2 and Endotype3.

b There is significant difference between Endotype1 and Endotype3.

c There is significant difference between Endotype1 and Endotype2.

DMARD, disease-modifying antirheumatic drug.

sDMARD, synthetic disease-modifying antirheumatic drug, including tacrolimus, cyclosporine A, cyclophosphamide, mycophenolate mofetil, Janus Kinase inhibitor, hydroxychloroquine, thalidomide, and iguratimod.

Biologic DMARDs include basiliximab, tocilizumab, and rituximab.

Tac, tacrolimus; CyA, cyclosporine A; CTX, cyclophosphamide; MMF, mycophenolate mofetil; Jaki, Janus Kinase inhibitor; HCQ, hydroxychloroquine; ILD, interstitial lung disease.

Low-dose steroid was defined as ≤ 0.5 mg/kg/day of prednisone; median-dose steroid was defined as > 0.5 and ≤ 1.0high dose of prednisone; high-dose steroid was defined as > 1 mg/kg/day of prednisone.

The P-value reflected the overall difference among the three groups.

The specific differences among every two groups were shown in **Supplementary Table 4**. Values in bold are statistically significant at p < 0.05.

### Circulating immune cell profiles alter in MDA5+ DM

**Figure 1:**
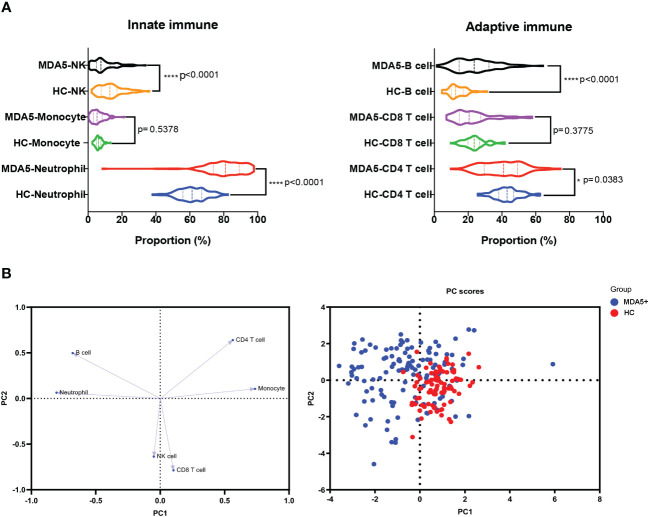
Immunological differences between MDA5+ DM and HC. **(A)** The differences in the compositions of the innate immune system (including neutrophil, monocyte, and NK cell) and the adaptive immune system (CD4^+^ T, CD8^+^ T, and CD19^+^B cell, namely B cell) between MDA5+ DM and HCs were shown. **(B)** The loading plot and the score plot of PCA visualized and verified the differences in immune cell profiles between MDA5+DM and HCs. The overall p-value is firstly marked (*P < 0.05 and ****P < 0.0001). Unpaired t-tests or Mann–Whitney U tests were used between two groups as appropriate.

We compared immunological distributions (including T, B and NK cells, neutrophils, and monocytes) between patients and HC, and found that the patients had a disrupted immunological landscape (**Figure 1A**). Specifically, these differences included a decrease in CD4^+^ T cells (39.98 vs 43.07%, *P* = 0.0383), NK cells (9.40 vs 13.56%, *P* < 0.0001), and an increase in neutrophils and B populations (80.58 vs 61.66%, *P* < 0.0001; 24.21 vs 13.76%, *P* < 0.0001, respectively) in patients with MDA5+ DM compared with HC. The alternations in peripheral immune cell subsets were further visualized via PCA (**Figure 1B**). PCA plots showed that the elevated neutrophils and B cells were the biggest immune cell changes.

### Unsupervised clustering analysis identified three MDA5+ DM endotypes

**Figure 2:**
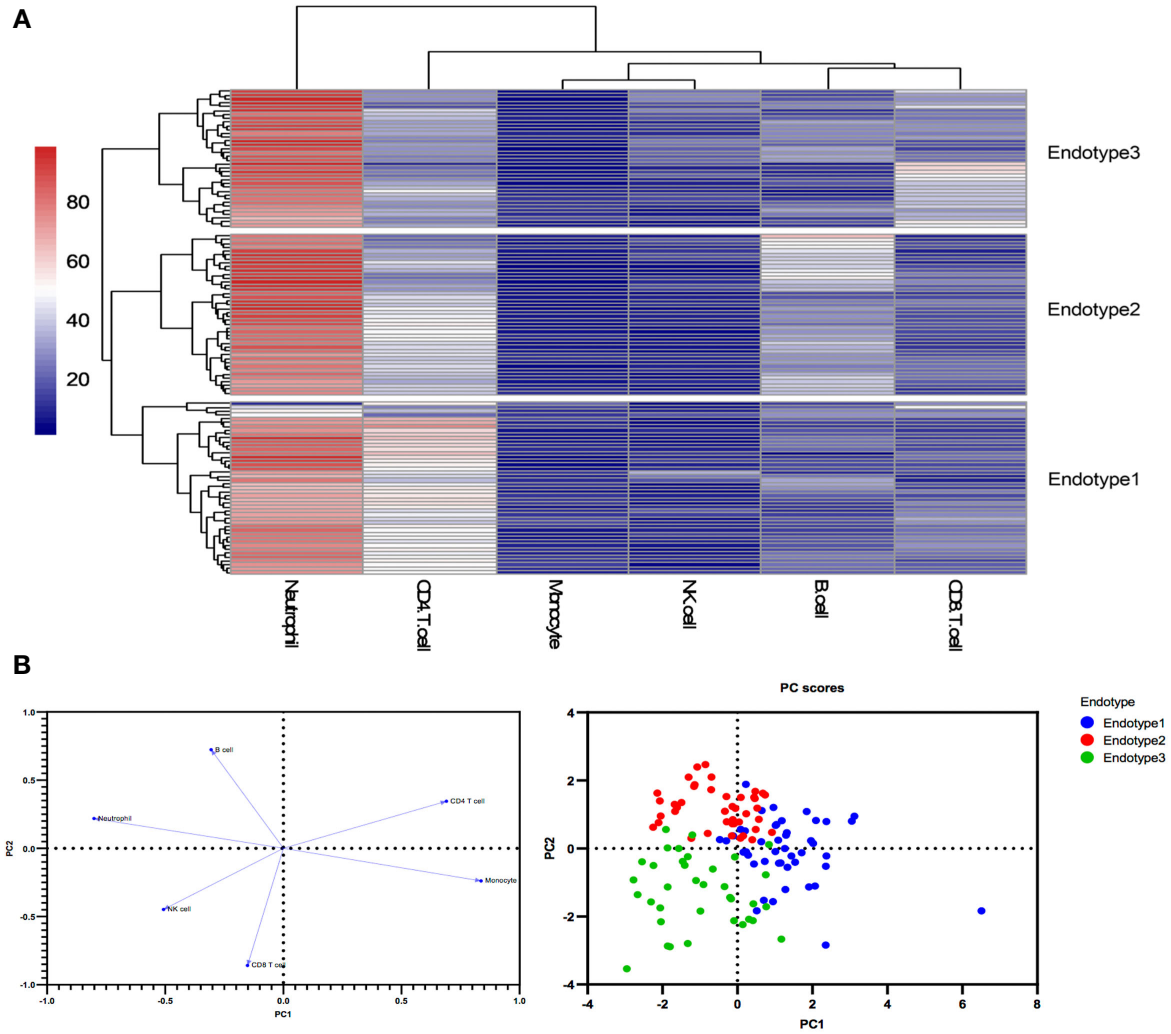
Peripheral immune cell profiles identify three endotypes in MDA5+ DM **(A)** Three endotypes of MDA5+ DM patients were divided through hierarchical clustering analysis. **(B)** The loading plot and the score plot of PCA visualized and verified the differences in immune cell profiles among the three MDA5+ DM endotypes.

Three MDA5+ DM endotypes (Endotype1, Endotype2, and Endotype3) were identified through hierarchical clustering analysis based on peripheral immune cell profiles. The results are shown in a combination of a dendrogram and a heatmap (**Figure 2A**). A two-dimensional PCA further confirmed that the three endotypes of MDA5+ DM patients had distinct immune cell profiles (**Figure 2B**).

**Figure 3:**
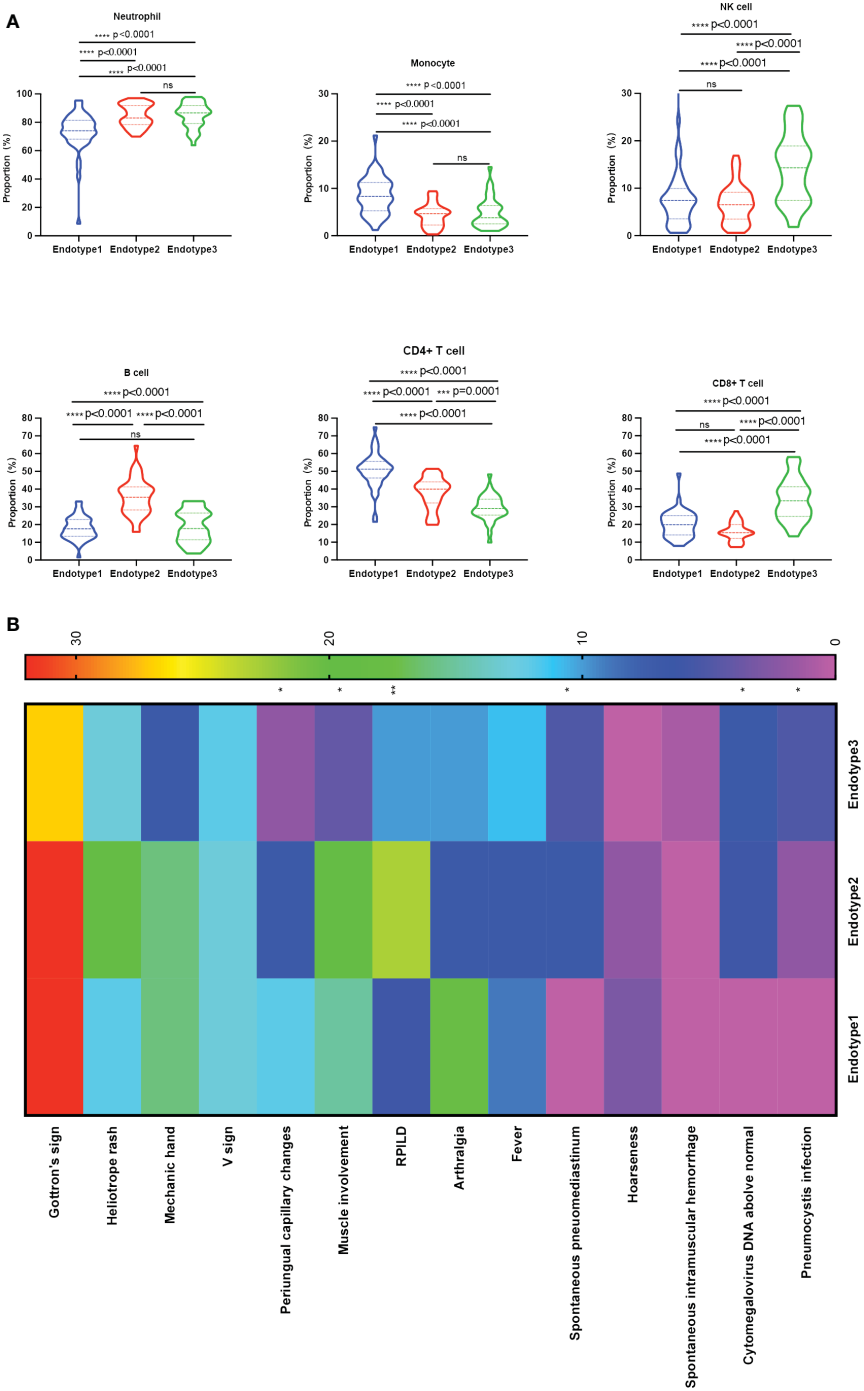
Comparisons among the three endotypes of patients with MDA5+ DM. **(A)** Violin plots showed the differences in the proportions of each immune cell subset among the clustered endotypes. **(B)** Clinical heterogeneity among the three endotypes was shown in the heatmap; The overall p-value is marked at the top of each image (*P < 0.05; **P < 0.005; ***P < 0.0005; ****P < 0.0001; ns, no significance). One-way ANOVA or Kruskal-Wallis H tests were used among three groups, while categorical variables were compared using chi-squared or Fisher’s exact tests as appropriate, followed by *post-hoc* tests for specific differences between every two groups.

The immunological compositions and changes of the three endotypes were displayed in **Figures 2A, B** and **Supplementary Table 2**. Among the three endotypes, Endotype1 (n = 45) had the highest percentages of CD4^+^ T cells and monocytes, and the lowest percentage of neutrophils (overall *P* < 0.0001 shown in top of the figure); Endotype2 (n = 42) had the highest percentage of B cells (overall *P* < 0.0001); while Endotype3 (n = 36) had the highest percentages of CD8^+^ T cells and NK cells, and the lowest percentage of CD4^+^ T cells (overall *P* < 0.0001), shown by **Figure 3A**. Cut-off values were determined for further clinical exploration. The cut-off for CD4^+^ T% was 42.4 (sensitivity 86.67%, specificity 82.05%), for B% was 26.05 (sensitivity 83.33%, specificity 82.72%) and for CD8^+^ T% was 24.05 (sensitivity 77.78%, specificity 82.76%) to identify patients from the other endotypes. ROC curves are shown in **Supplementary Figure 3**.

### Clinical characteristics differ in the MDA5+ DM endotypes

The three identified MDA5+ DM endotypes were further explored to determine whether different immune cell profiles lead to different clinical characteristics. The results are presented in **Figure 3B** and **Table 1**. And the specific differences between every two groups were shown in **Supplementary Table 3**. RPILD had the highest prevalence in Endotype2 (*P* < 0.001). Endotype1 had lower prevalence RPILD than Endotpye2 (*P* < 0.0001). Similarly, spontaneous pneumomediastinum occurred mostly in Endotype2 and no cases were recorded in Endotype1 (overall *P* = 0.005 among the three Endotypes). In addition, Endotype1 had higher incidence of periungual capillary changes than Endotype3, while Endotype2 had more patients with myalgia or muscle weakness than Endotpye3; the two clinical manifestations were less likely to be found in Endotype3 (overall *P* = 0.043 and 0.005, respectively; specific differences between every two groups seen in **Supplementary Table 3**). Only one case of spontaneous intramuscular hemorrhage occurred in Endotype3. Fewer males were found in Endotype1, when compared with Endotype3 (*P* = 0.0051). Laboratory test results are shown in **Table 1**. The lowest leukocyte counts were found in Endotype1 (overall *P* = 0.002). C-reactive protein level was highest in Endotype3 (overall *P* = 0.016). Cytomegalovirus infection and pneumocystis infection were mostly found in Endotype3, along with the lowest CD4/CD8 ratio (overall *P* = 0.003, 0.018, and < 0.001, respectively). Fibrinogen, creatinine, and urea levels also differed among endotypes, while γ-glutamyl transpeptidase (γ-GT) and alkaline phosphatase (ALP) levels were higher in Endotype2 (overall *P* = 0.024, 0.020, <0.001, 0.017, and 0.001, respectively). These results showed the clinical heterogeneity among the MDA5+ DM endotypes.

### Immune function fluctuations behind the MDA5+ DM endotypes

**Figure 4:**
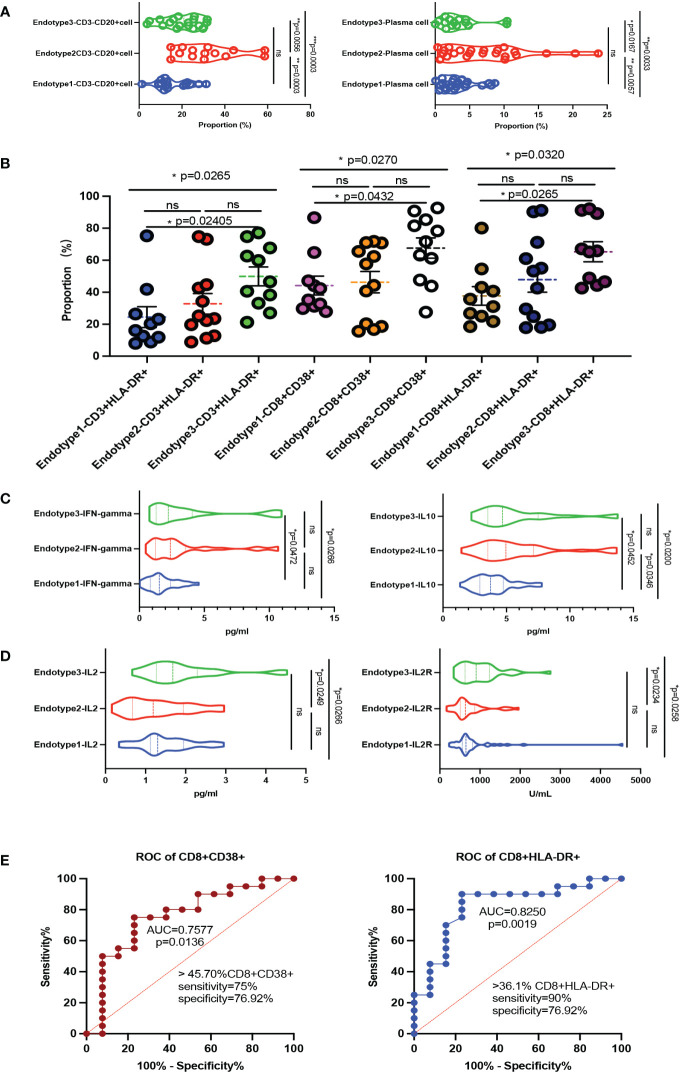
Immune function fluctuations behind the MDA5+ DM endotypes. **(A)** Violin plots showed CD3^-^CD20^+^ cells and plasma cells of B cell related subsets among the three endotypes. **(B)** The compositions of activated T cells differed in the three endotypes. **(C, D)** The main cytokines express levels (including IFN-gamma, IL-10, IL-2, IL-2R) among the three endotypes. **(E)** ROC curve of CD8^+^CD38^+^ cells and CD8^+^HLA-DR^+^ cells to distinguish infection or not among the MDA5+ DM; The overall p-value is firstly marked (*P < 0.05; **P < 0.005; ***P < 0.0005; ns, no significance). One-way ANOVA or Kruskal-Wallis H tests were used as appropriate among three groups followed by *post-hoc* tests for specific differences between every 2 groups.

Given the differences in the above immune cell profiles in patients and their impacts on clinical features, we further explore whether the function and status of these immune cells changed. We collected limited samples (n = 33, 10 in Endotype1, 12 in Endotype2, and 11 in Endotype3) in the MDA5+ DM for further analysis and found that the CD3^-^CD20^+^ cells and plasma cells in Endotype2 were significantly higher than those in the other two groups (all *P* < 0.05, shown in **Figure 4A**). To investigate the CD8^+^ T cell activation, we found CD8^+^CD38^+^ cells and CD8^+^HLA-DR^+^cells in the Endotype3 were significantly higher than Endotype1, but there was no statistically significant difference to the Endotype2 (**Figure 4B**). We further analyzed the related cytokines mainly by those immune cells, and found that the IFN-gamma and IL-10 in the Endotype3 were significantly higher than those in Endotype1, and the IL-2 and IL-2R in Endotype3 were higher than those in Endotype2 (all *P* < 0.05, **Figures 4C, D**). And the incidence of CMV and pneumocystis infection in the Endotpye3 group was significantly higher than that in the Endotype1. Therefore, under the complex immune changes, we explored whether those immune fluctuations in CD8^+^CD38^+^ and CD8^+^HLA-DR^+^ cells could distinguish infection among the endotypes. The cut-off of CD8^+^CD38^+^% was 45.7 (sensitivity 75%, specificity 76.92%) and CD8^+^HLA-DR^+^% was 36.1 (sensitivity 90%, specificity 76.92%). The ROC curves were shown in **Figure 4E**.

### Treatments and outcomes varied in the MDA5+ DM endotypes

**Figure 5:**
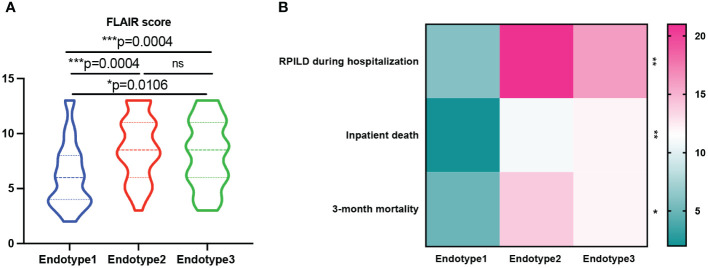
Prognosis of patients with MDA5+ DM. **(A)** FLAIR score was analyzed among the three endotypes. **(B)** Prognosis heterogeneity among the three endotypes was displayed in the heatmap; The overall p-value is firstly marked (*P < 0.05; **P < 0.005; ***P < 0.0005; ns, no significance). One-way ANOVA or Kruskal-Wallis H tests were used among three groups, while categorical variables were compared using chi-squared or Fisher’s exact tests as appropriate, followed by *post-hoc* tests for specific differences between every two groups.

Treatments and disease outcomes were further investigated to determine if the risk was stratified among the three MDA5+ DM endotypes (shown in **Table 2**, **Figures 5A, B**). And the specific differences between every two groups were shown in **Supplementary Table 4** and **Supplementary Figure 4**. The proportion of patients using a low dose of steroids (≤ 0.5 mg/kg/day of prednisone) was lower in Endotype2 than that in Endotype3 (*P* = 0.0120), while the proportion of patients taking a high dose of steroids (> 1 mg/kg/day of prednisone) was higher in Endotype2 than that in Endotype1 (*P* = 0.0062). Disease-modifying anti-rheumatic drugs, especially tacrolimus, were more frequently used in Endotype2 and less commonly in Endotype3 (overall *P* = 0.048 and 0.047, respectively). In addition, when using ILD progression, death during hospitalization, FLAIR score, and 3-month mortality to evaluate disease outcomes and prognosis, all these indicators showed that Endotype1 had the best outcomes with the least intensive treatment, lowest progression of ILD during hospitalization, inpatient death, 3-month mortality, and FLAIR score (**Figure 5**). Endotype2 and Endotype3 had higher incidence of inpatient ILD progression (*P* = 0.0004 and 0.0024, respectively) and were highly associated with 3-month mortality despite the most intensive treatment than that of Endotype1(P = 0.0185 and 0.0262, respectively). These results showed the prognostic heterogeneity of the MDA5+ DM endotypes.

## Discussion

MDA5+ DM is a distinct subtype of idiopathic inflammatory myopathies. The aetiology and pathogenesis of MDA5+ DM remain elusive, with a potential contributing role for T cells, B cells, neutrophils, and natural killer cells (2). Drugs targeting T cells (calcineurin inhibitors) and B cells (anti-CD20) have shown a certain role in clinical application (2, 13), so the classification based on immune cell subsets might provide a new perspective for the individualized treatment. In our current work, we have identified three disease endotypes based on peripheral immune cell profiles in an inpatient cohort. Endotypes based on clinical accessible data give us important references. Different clinical manifestations, laboratory results, and disease outcomes have also been revealed among the endotypes. The heterogeneity of the endotypes is multi-dimensional, with the potential to change clinicians’ perspectives on MDA5+ DM and inspire new research into the pathogenesis and therapy options for patients with different endotypes.

Among the identified endotypes, Endotype1 exhibited the best prognosis with the lowest incidence of death in 3 months, inpatient death, and inpatient ILD progression; it had the highest percentage of CD4^+^ T cells and monocytes, and the lowest percentage of neutrophils. A higher incidence of periungual capillary changes, a lower incidence of RPILD, and lower leukocyte counts were also features of this endotype. RPILD is known as an important predictor of mortality (22). Leukocyte count has been reported as an independent predictor of survival in CTD-ILD (23). Periungual erythema has also been found to be a protective predictor of survival in MDA5+ DM-ILD (24). We also observed in Endotype1 with the lowest mortality at 3 months had higher periungual erythema.

Endotype2 has the highest B cells percentage among the three endotype, showing notable treatment implications. Most patients had RPILD, spontaneous pneumomediastinum, muscle involvement, and inpatient ILD progression, despite the most intensive therapy. Levels of γ-GT, ALP, and urea-to-creatinine ratio were all higher in this endotype. As γ-GT was reported to be higher in dead patients with MDA5+ DM, and as liver dysfunction might be a clinical manifestation caused by activated macrophages in MDA5+ DM (25, 26), liver function monitoring should be recommended. Further, an increase in the urea to creatinine ratio (UCR) has been reported to reflect the state of catabolism and poor nutrition in persistent critical illness and other chronic diseases (27, 28). The increase of UCR observed in this endotype might be largely due to disease severity and poor overall condition of this endotype. As the endotype mainly featured an increase in the percentage of B cells, CD3^-^CD20^+^ cells, and plasma cells, B-cell -targeted therapy needs greater attention. Unfortunately, no such therapy was used in Endotype2 (shown in **Table 2**). A systematic review that presented information on 35 patients with MDA5+ DM who had received rituximab treatment found that 71.43% (25/35) of patients responded to treatment (29). The anti-CD20 drug seems to be promising, and other B-cell-targeted drugs including Bruton’s tyrosine kinase inhibitors are also worth investigating. Recently, a refractory case with a life-threatening RPILD in MDA5+ DM has been successfully treated with daratumumab, an anti-CD38-antibody (30). Single-cell RNA sequence on peripheral B cells also revealed that the B cell compartment was greatly activated with the terminal differentiation of antibody-secreting cell in active MDA5+ DM patients (13). More studies should be encouraged to explore the treatment potential of B-cell and CD38^+^ plasma-targeted therapies in MDA5+ DM, especially in the high B cell% endotype.

Endotype3 was characterized by the significantly elevated CD8^+^ T cells, having the highest level of CRP among the three groups, showing a poor prognosis that presented as higher RPILD, 3-month mortality than that in Endotype1. The expression levels of IL-2 and IL-2R, which are related to proliferative and activation of T cells (31, 32), were significantly elevated in Endotype3. Recent study also found that CD8^+^ T cell response is activated in MDA5+ DM, suggesting that CD8^+^ T cells could be the potential target (13). More cytomegalovirus and Pneumocystis infections were found in this endotype. Concomitant infection is a common complication of MDA5+ DM, which is also a potential trigger of MDA5-DM (2). Infections, such as viruses or fungi, have complex influence to immune system (2, 33). *In vivo* antigen-activated human T cells have been shown to be identified by a CD38 bright, HLA-DR^+^ phenotype after infection (34). Patients in Endotype3 showed the increased expression of IFN-gamma and the highest percentage of CD8^+^CD38^+^ cells and CD8^+^HLA-DR^+^ cells, which might be the complicated results of interactions between infection and primary disease. More researches are needed to explain the underlying mechanism and cause-and-effect relationship. In this state of MDA5+ DM, the cut-off of CD8^+^CD38^+^ cells and CD8^+^HLA-DR^+^ cells could distinguish ADM+ DM with or without infections, which might have a certain significance for clinical judgments and need more studies to validate. Uncontrolled expansion of CD8^+^ T cells producing IFN-gamma and other cytokines delay the termination step of the pro-inflammatory response (31). Studies have pointed out that CD38 is a surface marker of T cell exhaustion, which can inhibit the expression of cytotoxicity-related molecules through histone methyltransferase EZH2, thereby reducing the abilities of CD8^+^ T cell degranulation, perforin production and killing (35, 36), which increases the risk of infection in the body. These may further imply that both the disorder in the immune system and exposure to viral infection could trigger the deterioration of MDA5+ DM in different endotypes. Timely screening for concurrent infections and anti-infective therapies are crucial for better disease outcomes. The role of CD8^+^ T cells in MDA5+ DM requires further exploration.

This study has some limitations. Patients with missing data and those with <2500 lymphocytes for flow cytometry were not included. The need for prompt treatment and lack of untreated inpatients meant that the enrolled patients were already treated before sampling. Asymptomatic patients with no hospitalization history in our center could also be missed by the current study. It must be pointed out that our current work is mainly described in single-center, thus limiting the external validity of these findings. Further prospective studies are required to perform in other populations or diseases. However, this is a novel study that successfully identified distinct disease endotypes from immune cell profiles and fully explored their significance for the management of patients with MDA5 + DM.

## Conclusions

In conclusion, three disease endotypes were identified through hierarchical clustering analysis of the immune cell profiles of patients with MDA5+ DM. Clinical and prognostic differences were also confirmed among the endotypes, indicating the underlying heterogeneity. These results could help promote clinical stratification, pathogenesis investigations, and personalized treatment strategy or novel drug discovery targeting such key immune cell types in MDA5+ DM.

## Data availability statement

The original contributions presented in the study are included in the article/**Supplementary Material**. Further inquiries can be directed to the corresponding authors.

## Ethics statement

The studies involving humans were approved by Renji Hospital, Shanghai Jiao Tong University School of Medicine. The studies were conducted in accordance with the local legislation and institutional requirements. The participants provided their written informed consent to participate in this study.

## Author contributions

RG: Conceptualization, Data curation, Formal Analysis, Funding acquisition, Investigation, Methodology, Project administration, Resources, Supervision, Validation, Visualization, Writing – original draft, Writing – review & editing. YY: Data curation, Writing – original draft. LG: Resources, Writing – review & editing. XiL: Resources, Software, Writing – review & editing. YM: Data curation, Resources, Software, Writing – review & editing. XuL: Data curation, Funding acquisition, Investigation, Resources, Supervision, Writing – review & editing, Writing – original draft. LL: Funding acquisition, Investigation, Resources, Supervision, Writing – review & editing.

## References

[1] Fuzzi E Gatto M Zen M Franco C Zanatta E Ghirardello A. Anti-MDA5 dermatomyositis: an update from bench to bedside. Curr Opin Rheumatol. (2022) 34:365–73. doi: 10.1097/BOR.0000000000000908 PMC10810348 36094462

[2] Lu X Peng Q Wang G. Anti-MDA5 antibody-positive dermatomyositis: pathogenesis and clinical progress. Nat Rev Rheumatol. (2024) 20:48–62. doi: 10.1038/s41584-023-01054-9 38057474

[3] Wu W Guo L Fu Y Wang K Zhang D Xu W. Interstitial lung disease in anti-MDA5 positive dermatomyositis. Clin Rev Allergy Immunol. (2021) 60:293–304. doi: 10.1007/s12016-020-08822-5 33405101

[4] McPherson M Economidou S Liampas A Zis P Parperis K. Management of MDA-5 antibody positive clinically amyopathic dermatomyositis associated interstitial lung disease: A systematic review. Semin Arthritis Rheum. (2022) 53:151959. doi: 10.1016/j.semarthrit.2022.151959 35134633

[5] Selva-O’Callaghan A Romero-Bueno F Trallero-Araguás E Gil-Vila A Ruiz-Rodríguez JC Sánchez-Pernaute O. Pharmacologic treatment of anti-MDA5 rapidly progressive interstitial lung disease. Curr Treatm Opt Rheumatol. (2021) 7:319–33. doi: 10.1007/s40674-021-00186-x PMC8476986 34603940

[6] Tsuji H Nakashima R Hosono Y Imura Y Yagita M Yoshifuji H. Multicenter prospective study of the efficacy and safety of combined immunosuppressive therapy with high-dose glucocorticoid, tacrolimus, and cyclophosphamide in interstitial lung diseases accompanied by anti-melanoma differentiation-associated gene 5-positive dermatomyositis. Arthritis Rheumatol. (2020) 72:488–98. doi: 10.1002/art.41105 31524333

[7] Chen Z Wang X Ye S. Tofacitinib in amyopathic dermatomyositis-associated interstitial lung disease. N Engl J Med. (2019) 381:291–3. doi: 10.1056/NEJMc1900045 31314977

[8] Wu W Xu W Sun W Zhang D Zhao J Luo Q. Forced vital capacity predicts the survival of interstitial lung disease in anti-MDA5 positive dermatomyositis: a multi-centre cohort study. Rheumatol (Oxford). (2021) 61:230–9. doi: 10.1093/rheumatology/keab305 33764398

[9] Allenbach Y Uzunhan Y Toquet S Leroux G Gallay L Marquet A. Different phenotypes in dermatomyositis associated with anti-MDA5 antibody: Study of 121 cases. Neurology. (2020) 95:e70–8. doi: 10.1212/WNL.0000000000009727 PMC7371381 32487712

[10] McDowell PJ Heaney LG. Different endotypes and phenotypes drive the heterogeneity in severe asthma. Allergy. (2020) 75:302–10. doi: 10.1111/all.13966 31267562

[11] Angelini F Widera P Mobasheri A Blair J Struglics A Uebelhoer M. Osteoarthritis endotype discovery via clustering of biochemical marker data. Ann Rheum Dis. (2022) 81:666–75. doi: 10.1136/annrheumdis-2021-221763 35246457

[12] Deveza LA Nelson AE Loeser RF. Phenotypes of osteoarthritis: current state and future implications. Clin Exp Rheumatol. (2019) 37 Suppl 120:64–72. 31621574 PMC6936212

[13] Ye Y Chen Z Jiang S Jia F Li T Lu X. Single-cell profiling reveals distinct adaptive immune hallmarks in MDA5+ dermatomyositis with therapeutic implications. Nat Commun. (2022) 13:6458. doi: 10.1038/s41467-022-34145-4 36309526 PMC9617246

[14] Xu A Ye Y Fu Q Lian X Chen S Guo Q. Prognostic values of anti-Ro52 antibodies in anti-MDA5-positive clinically amyopathic dermatomyositis associated with interstitial lung disease. Rheumatol (Oxford). (2021) 60:3343–51. doi: 10.1093/rheumatology/keaa786 33331866

[15] Koichi Y Aya Y Megumi U Shunichi K Masafumi S Hiroaki M. A case of anti-MDA5-positive rapidly progressive interstitial lung disease in a patient with clinically amyopathic dermatomyositis ameliorated by rituximab, in addition to standard immunosuppressive treatment. Mod Rheumatol. (2017) 27:536–40. doi: 10.3109/14397595.2015.1014140 25698373

[16] Huang W Ren F Luo L Zhou J Huang D Pan Z. The characteristics of lymphocytes in patients positive for anti-MDA5 antibodies in interstitial lung disease. Rheumatol (Oxford). (2020) 59:3886–91. doi: 10.1093/rheumatology/keaa266 32535634

[17] Lv X Jin Y Zhang D Li Y Fu Y Wang S. Low circulating monocytes is in parallel with lymphopenia which predicts poor outcome in anti-melanoma differentiation-associated gene 5 antibody-positive dermatomyositis-associated interstitial lung disease. Front Med (Lausanne). (2021) 8:808875. doi: 10.3389/fmed.2021.808875 35111785 PMC8802832

[18] Jiang L Wang Y Peng Q Shu X Wang G Wu X. Serum YKL-40 level is associated with severity of interstitial lung disease and poor prognosis in dermatomyositis with anti-MDA5 antibody. Clin Rheumatol. (2019) 38:1655–63. doi: 10.1007/s10067-019-04457-w 30739212

[19] Zuo Y Ye L Chen F Shen Y Lu X Wang G. Different multivariable risk factors for rapid progressive interstitial lung disease in anti-MDA5 positive dermatomyositis and anti-synthetase syndrome. Front Immunol. (2022) 13:845988. doi: 10.3389/fimmu.2022.845988 35320936 PMC8936070

[20] Mammen AL Allenbach Y Stenzel W Benveniste O. 239th ENMC international workshop: classification of dermatomyositis, amsterdam, the Netherlands, 14-16 december 2018. Neuromuscul Disord. (2020) 30:70–92. doi: 10.1016/j.nmd.2019.10.005 31791867

[21] Lian X Zou J Guo Q Chen S Lu L Wang R. Mortality risk prediction in amyopathic dermatomyositis associated with interstitial lung disease: the FLAIR model. Chest. (2020) 158:1535–45. doi: 10.1016/j.chest.2020.04.057 32428508

[22] So J So H Wong VT Ho R Wu TY Wong PC. Predictors of rapidly progressive interstitial lung disease and mortality in patients with autoantibodies against melanoma differentiation-associated protein 5 dermatomyositis. Rheumatol (Oxford). (2022) 61:4437–44. doi: 10.1093/rheumatology/keac094 35157042

[23] Cao M Sheng J Qiu X Wang D Wang D Wang Y. Acute exacerbations of fibrosing interstitial lung disease associated with connective tissue diseases: a population-based study. BMC Pulm Med. (2019) 19:215. doi: 10.1186/s12890-019-0960-1 31727051 PMC6857302

[24] Li Y Li Y Wu J Miao M Gao X Cai W. Predictors of poor outcome of anti-MDA5-associated rapidly progressive interstitial lung disease in a chinese cohort with dermatomyositis. J Immunol Res. (2020) 2020:2024869. doi: 10.1155/2020/2024869 33299896 PMC7710415

[25] Gono T Kawaguchi Y Satoh T Kuwana M Katsumata Y Takagi K. Clinical manifestation and prognostic factor in anti-melanoma differentiation-associated gene 5 antibody-associated interstitial lung disease as a complication of dermatomyositis. Rheumatol (Oxford). (2010) 49:1713–9. doi: 10.1093/rheumatology/keq149 20498012

[26] Nagashima T Kamata Y Iwamoto M Okazaki H Fukushima N Minota S. Liver dysfunction in anti-melanoma differentiation-associated gene 5 antibody-positive patients with dermatomyositis. Rheumatol Int. (2019) 39:901–9. doi: 10.1007/s00296-019-04255-2 30790016

[27] Solimando AG Susca N Borrelli P Prete M Lauletta G Pappagallo F. Short-term variations in neutrophil-to-lymphocyte and urea-to-creatinine ratios anticipate intensive care unit admission of COVID-19 patients in the emergency department. Front Med (Lausanne). (2020) 7:625176. doi: 10.3389/fmed.2020.625176 33553217 PMC7854700

[28] Tufan F Yıldız A Dogan I Yıldız D Sevinir Ş. Urea to creatinine ratio: a forgotten marker of poor nutritional state in patients undergoing hemodialysis treatment. Aging Male. (2015) 18:49–53. doi: 10.3109/13685538.2014.908281 24702599

[29] He C Li W Xie Q Yin G. Rituximab in the treatment of interstitial lung diseases related to anti-melanoma differentiation-associated gene 5 dermatomyositis: A systematic review. Front Immunol. (2021) 12:820163. doi: 10.3389/fimmu.2021.820163 35116041 PMC8803653

[30] Holzer MT Nies JF Oqueka T Huber TB Kötter I Krusche M. Successful rescue therapy with daratumumab in rapidly progressive interstitial lung disease caused by MDA5-positive dermatomyositis. Chest. (2023) 163:e1–5. doi: 10.1016/j.chest.2022.08.2209 36628678

[31] Grom AA Horne A De Benedetti F. Macrophage activation syndrome in the era of biologic therapy. Nat Rev Rheumatol. (2016) 12:259–68. doi: 10.1038/nrrheum.2015.179 PMC5851441 27009539

[32] Jung JY Choi B Sayeed HM Suh CH Kim YW Kim HA. Characteristic patterns of HLA presentation and T cell differentiation in adult-onset Still’s disease. Int J Immunopathol Pharmacol. (2018) 32:2058738418791284. doi: 10.1177/2058738418791284 30052100 PMC6073833

[33] Rouse BT Sehrawat S. Immunity and immunopathology to viruses: what decides the outcome? Nat Rev Immunol. (2010) 10:514–26. doi: 10.1038/nri2802 PMC3899649 20577268

[34] Chandele A Sewatanon J Gunisetty S Singla M Onlamoon N Akondy RS. Characterization of human CD8 T cell responses in dengue virus-infected patients from India. J Virol. (2016) 90:11259–78. doi: 10.1128/JVI.01424-16 PMC5126381 27707928

[35] Tarragó MG Chini CCS Kanamori KS Warner GM Caride A de Oliveira GC. A potent and specific CD38 inhibitor ameliorates age-related metabolic dysfunction by reversing tissue NAD(+) decline. Cell Metab. (2018) 27:1081–1095.e10. doi: 10.1016/j.cmet.2018.03.016 29719225 PMC5935140

[36] Du J Wei L Li G Hua M Sun Y Wang D. Persistent high percentage of HLA-DR(+)CD38(high) CD8(+) T cells associated with immune disorder and disease severity of COVID-19. Front Immunol. (2021) 12:735125. doi: 10.3389/fimmu.2021.735125 34567001 PMC8458852

