# Supplementary material for: Disease-associated immune cell endotypes in anti-MDA5-positive dermatomyositis using unbiased hierarchical clustering
Source: Front Immunol. 2024 Mar 12;15:1349611. doi: 10.3389/fimmu.2024.1349611 (PMC10963492; doi:10.3389/fimmu.2024.1349611)
Supplement: Supplementary file 1 [file DataSheet_1.pdf]

Supplementary material

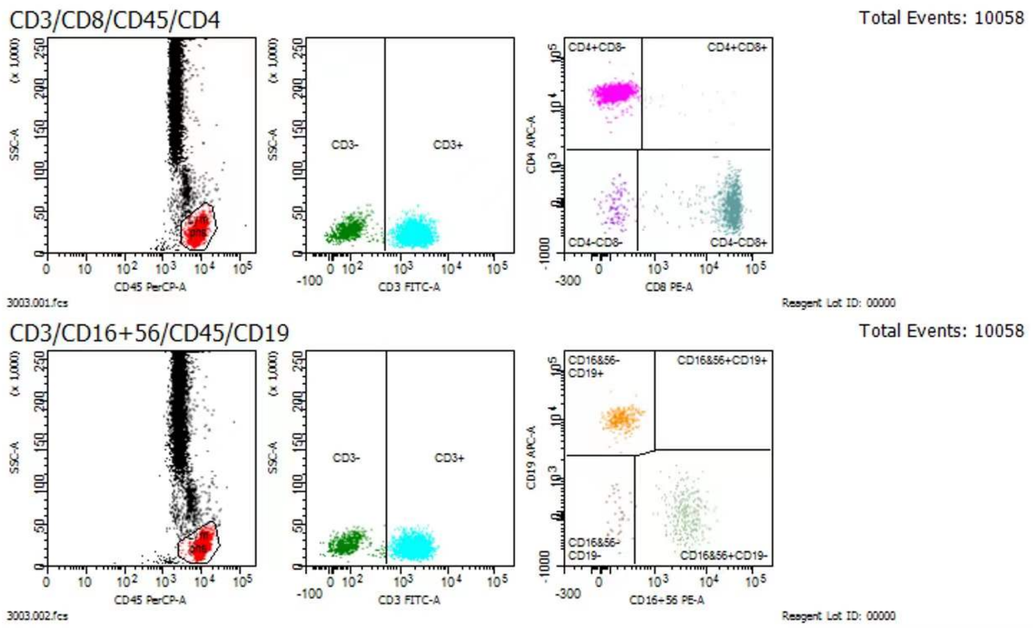

Supplementary Figure 1. The gating strategy of the flow cytometry analysis for T, B, NK cells. Lymphocytes were first gated. CD3-positive cells were then divided into CD4+ and CD8+ T cell groups, while CD3-negative cells were further separated into CD19+ B cells and CD16+/CD56+ NK cells.

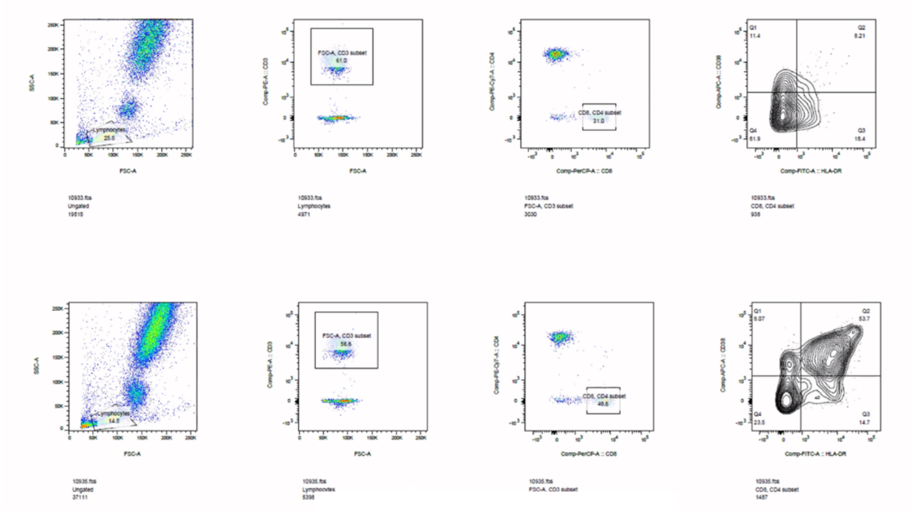

Supplementary Figure 2. The gating strategy of the flow cytometry analysis for activated CD8+ T cells.

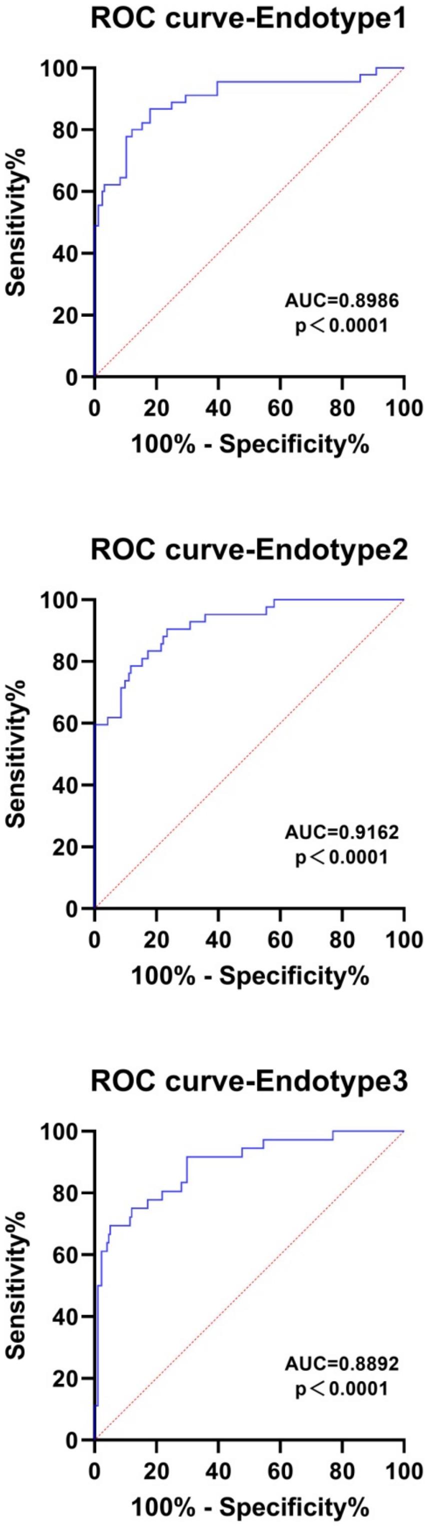

Supplementary Figure 3. The ROC curves of $CD4^+$ T cell%, B cell%, and $CD8^+$T cell% to identify patients from different endotypes.

Supplementary Figure 4 Immune function fluctuations behind the MDA5+ DM endotypes

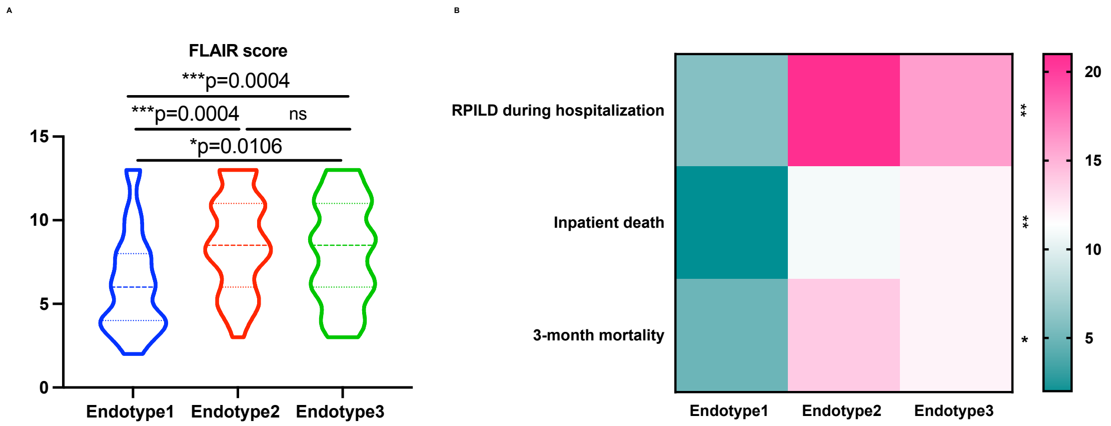

One-way ANOVA or Kruskal-Wallis H tests were used as appropriate among three groups followed by post-hoc tests for specific differences between every 2 groups. Categorical variables were compared using chi-squared or Fisher’s exact tests as appropriate.

RPILD during hospitalization:

Endotype1 vs Endotype2, p= 0.0004

Endotype1 vs Endotype3, p= 0.0024

Endotype2 vs Endotype3, p= 0.6555

Inpatients death:

Endotype1 vs Endotype2, p= 0.0059

Endotype1 vs Endotype3, p= 0.0008

Endotype2 vs Endotype3, p= 0.6194

3-month mortality:

Endotype1 vs Endotype2, p= 0.0185

Endotype1 vs Endotype3, p= 0.0262

Endotype2 vs Endotype3, p >0.9999

Supplementary Table 1 Treatment of the MDA5+ DM patients before sample collection.

Characteristics n(%)	Endotype1 (n = 45)	Endotype2 (n = 42)	Endotype3 (n = 36)	p-value
Low-dose steroid	24 (53.3)	17 (40.5)	22 (61.1)	
Median-dose steroid	19 (42.2)	22 (52.4)	14 (38.9)	0.286
High-dose steroid	2 (4.4)	3 (7.1)	0 (0)	
Tac	10 (22.2)	10 (23.8)	13 (36.1)	0.323
CyA	3 (6.7)	7 (16.7)	7 (19.4)	0.204
CTX	7 (15.6)	8 (19.0)	10 (27.8)	0.385
MMF	1 (2.2)	0 (0)	1 (2.8)	0.748
JAKi	2 (4.4)	4 (9.5)	3 (8.3)	0.684
Thalidomide	2 (4.4)	3 (7.1)	5 (13.9)	0.292
HCQ	14 (31.1)	10 (23.8)	14 (38.9)	0.356

Categorical variables were compared using chi-squared or Fisher's exact tests as appropriate.

Tac, tacrolimus; CyA, cyclosporine A; CTX, cyclophosphamide; MMF, mycophenolate mofetil; JAKi, Janus Kinase inhibitor; HCQ, hydroxychloroquine.

Low-dose steroid was defined as ≤ 0.5 mg/kg/day of prednisone; median-dose steroid was defined as > 0.5 and ≤ 1.0high dose of prednisone; high-dose steroid was defined as > 1 mg/kg/day of prednisone.

Supplementary Table 2 Differences of immune cell profiles among the three endotypes of the MDA5+ DM patients.

Immune cell	Endotype1 （n = 45）	Endotype2 （n = 42）	Endotype3 （n = 36）	*P*-value	
Neutrophil(%)[a]	73.23 ± 14.58	84.65 ± 7.87	85.03 ± 8.76	<0.0001	Endotype1 vs. Endotype2, p <0.0001<br>Endotype1 vs. Endotype3, p <0.0001<br>Endotype2 vs. Endotype3, p= 0.9870
Monocyte(%)[a]	8.53 ± 3.91	4.34 ± 2.39	4.84 ± 3.15	<0.0001	Endotype1 vs. Endotype2, p <0.0001<br>Endotype1 vs. Endotype3, p <0.0001<br>Endotype2 vs. Endotype3, p= 0.7805
NK cell(%)[b]	8.06 ± 6.45	6.71 ± 4.09	14.20 ± 7.14	<0.0001	Endotype1 vs. Endotype2, p= 0.5449<br>Endotype1 vs. Endotype3, p <0.0001<br>Endotype2 vs. Endotype3, p <0.0001
B cell(%)[c]	17.96 ± 6.48	35.61 ± 10.16	18.71 ± 9.05	<0.0001	Endotype1 vs. Endotype2, p <0.0001<br>Endotype1 vs. Endotype3, p= 0.9196<br>Endotype2 vs. Endotype3, p <0.0001
$CD4^+T$ cell(%)[d]	50.50 ± 9.88	37.83 ± 8.92	29.33 ± 7.32	<0.0001	Endotype1 vs. Endotype2, p <0.0001<br>Endotype1 vs. Endotype3, p <0.0001<br>Endotype2 vs. Endotype3, p= 0.0001
$CD8^+T$ cell(%)[b]	20.03 ± 7.58	15.82 ± 5.12	34.04 ± 11.85	<0.0001	Endotype1 vs. Endotype2, p= 0.0555<br>Endotype1 vs. Endotype3, p <0.0001<br>Endotype2 vs. Endotype3, p <0.0001

One-way ANOVA or Kruskal-Wallis H tests were used as appropriate among three groups followed by post-hoc tests for specific differences between every 2 groups.

[a]There is significant difference: Endotype1 vs Endtoype2, Endotype1 vs Endotype3

[b] There is significant difference: Endotype1 vs Endtoype3, Endotype2 vs Endotype3

[c] There is significant difference: Endotype1 vs Endtoype2, Endotype2 vs Endotype3

[d] There is significant difference between every two groups

Supplementary Table 3. Demographic and clinical characteristics of the three MDA5+ DM Endotypes

**Characteristics**	**All MDA5+DM (n=123)**	**Endotype 1 (n = 45)**	**Endotype2 (n = 42)**	**Endotype3 (n = 36)**	***overall P*-value**	
Age (years)	52.31 ± 12.07	52.89 ± 11.04	52.83 ± 11.71	50.97 ± 13.84	0.735	
Sex (M/F)[a]	32/91	6/39	15/27	11/25	**0.045**	Endotype1 vs. Endotype2, p= 0.1777<br>Endotype1 vs. Endotype3, p= 0.0051<br>Endotype2 vs. Endotype3, p= 0.1592
Clinical manifestations						
Shawl sign (n, %)	29 (23.6)	10 (22.2)	13 (31)	6 (16.7)	0.322	
Gottron's sign (n, %)	91 (73.9)	32 (71.1)	32 (76.2)	27 (75)	0.853	
Heliotrope rash (n, %)	44 (35.8)	12 (26.7)	19 (45.2)	13 (36.1)	0.196	
Mechanic hand (n, %)	39 (31.7)	16 (35.6)	16 (38.1)	7 (19.4)	0.165	
V sign (n, %)	38 (30.9)	13 (28.9)	13 (31)	12 (33.3)	0.912	
Periungual capillary changes (n, %)[a]	21 (17.1)	12 (26.7)	7 (16.7)	2 (5.5)	**0.043**	Endotype1 vs. Endotype2, p= 0.3062<br>Endotype1 vs. Endotype3, p= 0.0169<br>Endotype2 vs. Endotype3, p= 0.1658
Myalgia or muscle weakness (n, %)[b]	38 (30.9)	15 (33.3)	19 (45.3)	4 (11.1)	**0.005**	Endotype1 vs. Endotype2, p= 0.2794<br>Endotype1 vs. Endotype3, p= 0.0331<br>Endotype2 vs. Endotype3, p= 0.0011
RPILD (n, %)[c]	39 (31.7)	6 (13.3)	23 (54.8)	10 (27.8)	< **0.001**	Endotype1 vs. Endotype2, p <0.0001<br>Endotype1 vs. Endotype3, p= 0.1596<br>Endotype2 vs. Endotype3, p= 0.0218
Arthralgia (n, %)	35 (28.5)	18 (40)	7 (16.7)	10 (27.8)	0.054	
Fever (n, %)	27 (21.9)	9 (20)	7 (16.7)	11 (30.6)	0.310	

Spontaneous pneumomediastinum (n, %)[d]	12 (9.8)	0 (0)	7 (16.7)	5 (13.9)	**0.005**	Endotype1 vs. Endotype2, p= 0.0046<br>Endotype1 vs. Endotype3, p= 0.0147<br>Endotype2 vs. Endotype3, p= 0.7647
Hoarseness (n, %)	5 (4.1)	3 (6.7)	2 (4.8)	0 (0)	0.376	
Spontaneous intramuscular hemorrhage (n, %)	1 (0.8)	0 (0)	0 (0)	1 (2.8)	0.293	
Days of follow-up (days)	245.00 ± 232.20	275.00 ± 331.40	208.00 ± 287.8	250.60 ± 355.20	0.626	
Laboratory results						
Leukocyte counts (×10^9/L)[e]	7.20 ± 3.38	5.94 ± 2.64	8.21 ± 3.67	7.59 ± 3.43	**0.002**	Endotype1 vs. Endotype2, p= 0.0042<br>Endotype1 vs. Endotype3, p= 0.0647<br>Endotype2 vs. Endotype3, p= 0.6771
Ferritin (μg/L)	1142 ± 1178	1048.10 ± 931.12	1391.92 ± 1662.5	968.04 ± 623.69	0.229	
LDH (U/L)	357.70 ± 182.70	318.20 ± 106.26	390.55 ± 245.36	368.58 ± 167.89	0.167	
CRP (mg/L)[b]	10.95 ± 20.10	8.24 ± 21.42	9.22 ± 10.65	16.61 ± 26.13	**0.016**	Endotype1 vs. Endotype2, p= 0.9812<br>Endotype1 vs. Endotype3, p= 0.0272<br>Endotype2 vs. Endotype3, p= 0.0471
ESR (mm/h)	38.63 ± 23.08	37.20 ± 24.47	38.36 ± 22.25	40.75 ± 22.72	0.788	
Fibrinogen (g/L)[a]	3.33 ± 1.03	3.04 ± b0.80	3.35 ± 1.07	3.67 ± 1.17	**0.024**	Endotype1 vs. Endotype2, p= 0.3322<br>Endotype1 vs. Endotype3, p= 0.0178<br>Endotype2 vs. Endotype3, p= 0.3477
ALT (U/L)	75.16 ± 99.18	77.62 ± 95.13	96.00 ± 132.01	47.78 ± 34.61	0.157	
γGT (U/L)[e]	134.00 ± 212.70	78.47 ± 76.43	216.61 ± 333.3	114.46 ± 103.94	**0.017**	Endotype1 vs. Endotype2, p= 0.0071<br>Endotype1 vs. Endotype3, p= 0.7218

						Endotype2 vs. Endotype3, p= 0.0859
ALP (U/L)[e]	91.42 ± 46.51	74.80±20.23	108.27 ± 58.03	95.71 ± 49.09	**0.001**	Endotype1 vs. Endotype2, p= 0.0020<br>Endotype1 vs. Endotype3, p= 0.0982<br>Endotype2 vs. Endotype3, p= 0.4418
CK (U/L)	115.60 ± 230.8	89.78 ± 96.50	140.4 ± 259.8	121.9 ± 309.4	0.396	
Cr (µmol/L)[a]	51.47 ± 20.23	46.19 ± 9.77	51.28 ± 15.32	58.30 ± 30.79	**0.020**	Endotype1 vs. Endotype2, p= 0.4564<br>Endotype1 vs. Endotype3, p= 0.0194<br>Endotype2 vs. Endotype3, p= 0.2657
Urea (mmol/L)[d]	6.17 ± 2.99	4.93 ± 1.36	7.08 ± 3.30	6.81 ± 3.64	< **0.001**	Endotype1 vs. Endotype2, p= 0.0050<br>Endotype1 vs. Endotype3, p= 0.0115<br>Endotype2 vs. Endotype3, p= 0.9885
UCR[e]	0.12 ± 0.04	0.11 ± 0.31	0.14 ± 0.05	0.12 ± 0.38	**0.049**	Endotype1 vs. Endotype2, p=0.0382<br>Endotype1 vs. Endotype3, p=0.4583<br>Endotype2 vs. Endotype3, p=0.4752
CD4/CD8[b]	2.29 ± 1.40	3.00 ± 1.46	2.66 ± 1.12	1.00 ± 0.46	< **0.001**	Endotype1 vs. Endotype2, p= 0.3375<br>Endotype1 vs. Endotype3, p <0.0001<br>Endotype2 vs. Endotype3, p <0.0001
Cytomegalovirus DNA above normal (n, %)[d]	13 (10.6)	0 (0)	6 (14.3)	7 (19.4)	**0.003**	Endotype1 vs. Endotype2, p=0.0104<br>Endotype1 vs. Endotype3, p=0.0024<br>Endotype2 vs. Endotype3, p=0.5599
Pneumocystis infection (n%)[a]	7 (5.7)	0 (0)	2 (4.8)	5 (13.9)	**0.018**	Endotype1 vs. Endotype2, p=0.2302<br>Endotype1 vs. Endotype3, p=0.0147<br>Endotype2 vs. Endotype3, p=0.2387

One-way ANOVA or Kruskal-Wallis H tests were used among three groups, while categorical variables were compared using chi-squared or Fisher's exact tests as appropriate, followed by post-hoc tests for specific differences between every two groups.

[a] There is significant difference between Endotype1 and Endotype3

[b] There is significant difference: Endotype1 vs Endtoype3, Endotype2 vs Endotype3

[c] There is significant difference: Endotype1 vs Endtoype2, Endotype2 vs Endotype3

[d] There is significant difference: Endotype1 vs Endtoype2, Endotype1 vs Endotype3

[e] There is significant difference between Endotype1 and Endotype2

RPILD, rapidly progressive interstitial lung disease; LDH, lactate dehydrogenase; CRP, C-reactive protein; ESR, erythrocyte sedimentation rate; ALT, alanine transaminase; γGT, γ-glutamyl transpeptidase; ALP, alkaline phosphatase; CK, creatine kinase; Cr, creatinine; UCR, urea to creatinine ratio; NMR, neutrophil to monocyte ratio

The *P*-value reflected the difference among the three groups.

Supplementary Table 4 Inpatient treatment and prognosis of the MDA5+ DM patients.

Characteristics	All MDA5+DM (n=123)	Endotype1 (n = 45)	Endotype2 (n = 42)	Endotype3 (n = 36)	*overall* ***P*****-value**	
Inpatient Treatments						
Low-dose steroid[a]	22 (17.9)	6 (13.3)	4 (9.5)	12 (33.3)	**0.002**	Endotype1 vs. Endotype2, p=0.7402<br>Endotype1 vs. Endotype3, p=0.0578<br>Endotype2 vs. Endotype3, p=0.0120
Median-dose steroid[b]	86 (69.9)	38 (84.4)	29 (69)	19 (52.8)		Endotype1 vs. Endotype2, p=0.1260<br>Endotype1 vs. Endotype3, p=0.0030<br>Endotype2 vs. Endotype3, p=0.1662
High-dose steroid[c]	15 (12.2)	1 (2.2)	9 (21.4)	5 (13.9)		Endotype1 vs. Endotype2, p= 0.0062<br>Endotype1 vs. Endotype3, p= 0.0834<br>Endotype2 vs. Endotype3, p= 0.5554
DMARDs[a]	103 (83.7)	38 (84.4)	39 (92.9)	26 (72.2)	**0.048**	Endotype1 vs. Endotype2, p= 0.3170<br>Endotype1 vs. Endotype3, p= 0.2720<br>Endotype2 vs. Endotype3, p= 0.0300
Biologic DMARDs	18 (14.6)	5 (11.1)	7 (16.7)	6 (16.7)	0.703	
Tac[a]	66 (53.7)	21 (46.7%)	29 (69.0%)	16 (44.4%)	**0.047**	Endotype1 vs. Endotype2, p= 0.0506<br>Endotype1 vs. Endotype3, p >0.9999<br>Endotype2 vs. Endotype3, p= 0.0389
CyA	22 (17.9)	10 (22.2%)	7(16.7%)	5 (13.9%)	0.603	
CTX	4 (3.3)	3 (6.7%)	0	1 (2.8%)	0.205	
MMF	2 (1.6)	1 (2.2%)	0 (0%)	1 (2.8%)	0.748	
Jaki	22 (17.9)	9 (20%)	9 (21.4%)	4 (11.1%)	0.445	
Thalidomide	10 (8.1)	3 (6.7%)	6 (14.3%)	1 (2.8%)	0.199	
Basiliximab	13 (10.6)	4 (8.9%)	6 (14.3%)	3 (8.3%)	0.709	
Rituximab	2 (1.6))	1 (2.2%)	0 (0%)	1 (2.8%)	0.748	

Tocilizumab	4 (3.3)	1(2.2%)	1 (2.4%)	2 (5.6%)	0.681	
Iguratimod	11 (8.9)	2 (4.4%)	4 (9.5%)	5 (13.9%)	0.323	
HCQ	10 (8.1)	2 (4.4%)	4 (9.5%)	4 (11.1%)	0.499	
No.of sDMARDS ≥ 2	39 (31.7)	11 (24.4)	19 (45.2)	9 (25)	0.067	
Prognosis						
Progression of ILD[bc] during hospitalization	43 (34.9)	6 (13.3)	21 (50)	16 (44.4)	**0.001**	Endotype1 vs. Endotype2, p= 0.004<br>Endotype1 vs. Endotype3, p= 0.024<br>Endotype2 vs. Endotype3, p= 0.6555
Inpatient death[bc]	25 (20.3)	2 (4.4)	11 (26.2)	12 (33.3)	**0.003**	Endotype1 vs. Endotype2, p= 0.0059<br>Endotype1 vs. Endotype3, p= 0.0008<br>Endotype2 vs. Endotype3, p= 0.6194
3-month mortality[bc]	31 (25.2)	5 (11.1)	14 (33.3)	12 (33.3)	**0.024**	Endotype1 vs. Endotype2, p= 0.0185<br>Endotype1 vs. Endotype3, p= 0.0262<br>Endotype2 vs. Endotype3, p= >0.9999

One-way ANOVA or Kruskal-Wallis H tests were used among three groups, while categorical variables were compared using chi-squared or Fisher's exact tests as appropriate, followed by post-hoc tests for specific differences between every two groups.

[a] There is significant difference between Endotype2 and Endotype3

[b] There is significant difference between Endotype1 and Endotype3

[c] There is significant difference between Endotype1 and Endotype2

DMARD, disease-modifying antirheumatic drug

sDMARD, synthetic disease-modifying antirheumatic drug, including tacrolimus, cyclosporine A, cyclophosphamide, mycophenolate mofetil, Janus Kinase inhibitor, hydroxychloroquine, thalidomide, and iguratimod

Biologic DMARDs include basiliximab, tocilizumab, and rituximab

Tac, tacrolimus; CyA, cyclosporine A; CTX, cyclophosphamide; MMF, mycophenolate mofetil; Jaki, Janus Kinase inhibitor; HCQ, hydroxychloroquine; ILD,

interstitial lung disease

Low-dose steroid was defined as ≤ 0.5 mg/kg/day of prednisone; median-dose steroid was defined as > 0.5 and ≤ 1.0high dose of prednisone; high-dose steroid was defined as > 1 mg/kg/day of prednisone.

The *P*-value reflected the overall difference among the three groups.
